# CD8+ NKs as a potential biomarker of complete response and survival with lenalidomide plus R-GDP in the R2-GDP-GOTEL trial in recurrent/refractory diffuse large B cell lymphoma

**DOI:** 10.3389/fimmu.2024.1293931

**Published:** 2024-02-26

**Authors:** Lourdes Hontecillas-Prieto, Daniel J. García-Domínguez, Natalia Palazón-Carrión, Alejandro Martín García-Sancho, Esteban Nogales-Fernández, Carlos Jiménez-Cortegana, María L. Sánchez-León, Silvia Silva-Romeiro, Rocío Flores-Campos, Fernando Carnicero-González, Eduardo Ríos-Herranz, Fátima de la Cruz-Vicente, Guillermo Rodríguez-García, Rubén Fernández-Álvarez, Natividad Martínez-Banaclocha, Josep Gumà-Padrò, José Gómez-Codina, Antonio Salar-Silvestre, Delvys Rodríguez-Abreu, Laura Gálvez-Carvajal, Jorge Labrador, María Guirado-Risueño, Mariano Provencio-Pulla, Margarita Sánchez-Beato, Lejeune Marylene, Tomás Álvaro-Naranjo, María Casanova-Espinosa, Antonio Rueda-Domínguez, Víctor Sánchez-Margalet, Luis de la Cruz-Merino

**Affiliations:** ^1^ Clinical Biochemistry Service, Virgen Macarena University Hospital, University of Seville, Seville, Spain; ^2^ Department of Medical Biochemistry and Molecular Biology and Immunology, Medical School, Virgen Macarena University Hospital, University of Seville, Seville, Spain; ^3^ Institute of Biomedicine of Seville, Virgen Macarena University Hospital, CSIC, University of Seville, Seville, Spain; ^4^ Clinical Oncology Service, Hospital Universitario Virgen Macarena, University of Seville, Seville, Spain; ^5^ Department of Medicine, University of Seville, Seville, Spain; ^6^ Department of Hematology, Hospital Universitario de Salamanca, IBSAL, CIBERONC, University of Salamanca, Salamanca, Spain; ^7^ Department of Hematology, Hospital San Pedro de Alcántara de Cáceres, Cáceres, Spain; ^8^ Department of Hematology, Hospital Universitario de Valme, Seville, Spain; ^9^ Department of Hematology, Hospital Universitario Virgen del Rocío, Seville, Spain; ^10^ Department of Hematology, Cabueñes Hospital, Gijón, Spain; ^11^ Oncology Dept., Dr. Balmis General University Hospital, Alicante Institute for Health and Biomedical Research (ISABIAL), Alicante, Spain; ^12^ Department of Clinical Oncology, Hospital Universitari Sant Joan de Reus URV, IISPV, Reus, Spain; ^13^ Department of Clinical Oncology, Hospital Universitario La Fé, Valencia, Spain; ^14^ Department of Hematology, Hospital del Mar, Barcelona, Spain; ^15^ Department of Clinical Oncology, Hospital Universitario Insular, Las Palmas de Gran Canaria, Spain; ^16^ Department of Medical Oncology Intercenter Unit, Regional and Virgen de la Victoria University Hospitals, IBIMA, Málaga, Spain; ^17^ Department of Hematology, Research Unit, Hospital Universitario de Burgos, Burgos, Spain; ^18^ Department of Clinical Oncology, Hospital General Universitario de Elche, Elche, Spain; ^19^ Department of Medical Oncology, Hospital Universitario Puerta de Hierro-Majadahonda, Facultad de Medicina, Universidad Autónoma de Madrid, IDIPHISA, Madrid, Spain; ^20^ Department of Medical Oncology, Lymphoma Research Group, Hospital Universitario Puerta de Hierro-Majadahonda, IDIPHISA, CIBERONC, Madrid, Spain; ^21^ Department of Pathology, Plataforma de Estudios Histológicos, Citológicos y de Digitalización, Hospital de Tortosa Verge de la Cinta, IISPV, URV, Tortosa, Tarragona, Spain; ^22^ Department of Pathology, Hospital de Tortosa Verge de la Cinta, Catalan Institute of Health, Institut d’Investigació Sanitària Pere Virgili (IISPV), Tortosa, Tarragona, Spain; ^23^ Department of Hematology/Clinical Oncology, Hospital Costa del Sol, Marbella, Spain

**Keywords:** DLBCL, B cell lymphoma, recurrent/refractory disease, immune system, natural killer, CD8+ NK, biomarker, R2-GDP-GOTEL

## Abstract

**Background:**

Diffuse large B cell lymphoma (DLBCL) is the most common non-Hodgkin lymphoma worldwide. DLBCL is an aggressive disease that can be cured with upfront standard chemoimmunotherapy schedules. However, in approximately 35-40% of the patients DLBCL relapses, and therefore, especially in this setting, the search for new prognostic and predictive biomarkers is an urgent need. Natural killer (NK) are effector cells characterized by playing an important role in antitumor immunity due to their cytotoxic capacity and a subset of circulating NK that express CD8 have a higher cytotoxic function. In this substudy of the R2-GDP-GOTEL trial, we have evaluated blood CD8+ NK cells as a predictor of treatment response and survival in relapsed/refractory (R/R) DLBCL patients.

**Methods:**

78 patients received the R2-GDP schedule in the phase II trial. Blood samples were analyzed by flow cytometry. Statistical analyses were carried out in order to identify the prognostic potential of CD8+ NKs at baseline in R/R DLBCL patients.

**Results:**

Our results showed that the number of circulating CD8+ NKs in R/R DLBCL patients were lower than in healthy donors, and it did not change during and after treatment. Nevertheless, the level of blood CD8+ NKs at baseline was associated with complete responses in patients with R/R DLBCL. In addition, we also demonstrated that CD8+ NKs levels have potential prognostic value in terms of overall survival in R/R DLBCL patients.

**Conclusion:**

CD8+ NKs represent a new biomarker with prediction and prognosis potential to be considered in the clinical management of patients with R/R DLBCL.

**Clinical trial registration:**

https://www.clinicaltrialsregister.eu/ctr-search/search?query=2014-001620-29 EudraCT, ID:2014-001620-29.

## Introduction

Lymphoproliferative diseases comprise a diverse and heterogeneous group of malignancies (1). Diffuse large B-cell lymphoma (DLBCL) is the most common non-Hodgkin lymphoma (NHL) subtype, accounting 30%-40% of lymphoid malignancies (2). Chemoimmunotherapy with R-CHOP (rituximab, cyclophosphamide, doxorubicin, vincristine, and prednisone) and R-CHOP-like schedules remains the upfront standard of care in DLBCL. However, one-third of DLBCL patients will relapse having a poor outcome, especially the cases with refractory disease to frontline or subsequent therapies (2). Although new strategies as chimeric antigen receptor T-cells (CART), bispecific monoclonal antibodies and new combinations with anti-CD19 (tafasitamab) plus lenalidomide or antibody drug conjugates (polatuzumab) plus bendamustine and rituximab are increasing the therapeutic armamentarium in relapsed/refractory (R/R) DLBCL (3), the search of new reliable predictive and prognostic biomarkers that could guide clinical management and eventually point to new therapeutic targets is extremely relevant and necessary.

Antitumor immune cells in peripheral blood have gained increasing relevance and interest, particularly natural killer cells (NKs). NK population is responsible for immune surveillance and represents major component of innate immunity against virus infected cells or malignant cells (4). NKs are effector cells characterized by exerting strong cytotoxicity against tumor cells and play an important role in the efficacy of rituximab-based therapy due to their ability to induce antibody-dependent cell cytotoxicity (ADCC) (5, 6). In DLBCL patients, some studies have evaluated NKs in peripheral blood. Indeed, NK cell count was associated with response and event free survival independently of adverse age-adjusted International Prognostic Index (7). In this line, low baseline NK cell count has also been associated with shorter progression-free survival (8). In these studies, NKs were defined as CD3-CD16 + 56+. Nevertheless, NKs express a large number of surface antigens (9); and, consequently, many subsets of NKs have been described in human peripheral blood (10, 11). One of the most promising and underexplored subsets of NKs in cancer are those that express CD8+ at lower levels than T cells (12, 13). The CD8 expression on NKs seems to be associated with a higher cytotoxic function compared with CD8- NK cells in healthy humans (14, 15), and in avian CD8 identify the lytic NK (16). Moreover, CD8+ NKs are capable of sequential lysis of multiple target cells (12). Regarding their relevance in human diseases, CD8+ NKs have been found to exert a suppressive effect in relapsing remitting multiple sclerosis (13). In chronic human immunodeficiency virus (HIV) patients an initial loss of this subset of NKs has been described, followed by a phenotypic change in CD8- NKs to become CD8+ in the progression of the disease (9). Moreover, high CD8+ NKs have been associated with slower disease progression exhibiting a more functional profile (17). Finally, it has been reported that CD8+ NKs mediate the autologous cytotoxicity of myeloid leukemic cells from patients in clinical remission after autologous stem-cell transplantation (18) and of acute myeloid leukemia patients in complete remission after chemotherapy alone *in vitro* (19).

Recently in the GOTEL clinical trial in R/R DLBCL, we found that the number of circulating myeloid-derived suppressor cells (MDSCs) after the third cycle of treatment was a good immunological biomarker associated with both survival (2, 20) and clinical benefit (21). However, the number of basal circulating MDSCs did not predict survival or clinical benefit. As a result, we have continued to search for blood biomarkers of treatment response in this clinical trial. As CD8+ NK cells seem to have a high cytotoxic function against tumor cells, we analyzed this subset of NKs in patients treated with R-GDP plus lenalidomide in the R2-GDP-GOTEL phase II trial and evaluated their prognostic impact at baseline. The results obtained showed that CD8+ NKs, but not CD8- NKs, are associated with complete responses and, more importantly, with overall survival (OS), representing a promising new biomarker with prediction and prognosis potential in R/R DLBCL.

## Materials and methods

### Study design

79 patients diagnosed with R/R DLBCL were enrolled in this multicenter (78 patients were finally considered in the intention to treat (ITT) analysis due to the voluntary withdrawal of one patient), open-label, single-arm R2-GDP-GOTEL phase II clinical trial study (EudraCT Number: 2014-001620-29) (21). The main baseline characteristics of the patients are summarized in **Supplementary Table 1** and the progress of patients through the trial are summarized in **Supplementary Figure 1A**. The study was conducted in compliance with the International Ethical Guidelines for Biomedical Research Involving Human Subjects, the Declaration of Helsinki, good clinical practice guidelines, and local laws. The study protocol and any subsequent amendments were approved by Seville Provincial Ethics Committee for Research with Drug.

### Lenalidomide plus R-GDP treatment

R/R DLBCL patients received the R2-GDP schedule, based on lenalidomide in combination with R-GDP. After a first run-in phase period the following schedule was administered: intravenous rituximab 375 mg/m^2^ on day (D)1, intravenous cisplatin 60 mg/m^2^ D1, intravenous gemcitabine 750 mg/m^2^ D1 and D8, oral dexamethasone 20 mg D1–3, subcutaneous granulocyte colony stimulating factor (G-CSF) 30 million units international (MUI) D2–6 and D9–14 in combination with oral lenalidomide 10 mg D1–14, in cycles every 3 weeks. If after the 3^rd^ cycle there was no progression of disease, a maximum of 6 induction cycles were administered. Patients that reached clinical benefit after at least 3 cycles of treatment could enter a maintenance phase with lenalidomide 10 mg (or the last dose administered in the induction phase) D1–21 in cycles every 4 weeks. The maintenance phase was intended to continue until progression, unacceptable toxicity, patient voluntary withdrawal, or when two positron emission tomography (PET) confirmed metabolic complete response after 2 years of treatment.

### Response evaluation and outcome

Evaluation of the response to treatment allowed us to define patients in complete response (CR), partial response (PR), stable disease (SD) or patients with progression of the disease (PD). Tumor response was evaluated according to the International Working Group Criteria (22) using computed tomography after the third induction cycle and PET in the following 4 weeks after the last cycle of the induction phase, and the response to treatment was calculated as Best Overall Response.

### Immunophenotyping

NKs and other immune cells were studied in peripheral blood from R/R DLBCL patients during the R2-GDP-GOTEL study at three time-points: basal, cycle 3 and end of induction (EOI). Blood samples were collected in EDTA-K3 tubes and cell populations were determined by flow cytometry analysis using the BD FACSCanto II™ flow cytometry system with the monoclonal antibodies (mAbs) and protocols recommended by Becton Dickinson Immunocytometry Systems (BDIS, San Jose, CA, USA). mAbs are listed in **Supplementary Table 2** and the phenotypes for immune cell studies are described in **Supplementary Table 3**.

Lymphocyte subpopulations were analyzed by BD Multi-test 6-Color TBNK (Becton Dickinson). NK cells were gated by selecting the CD3- and CD16 + 56+ cells, and then analyzed as CD8- and CD8+ (**Supplementary Figure 1B**).

### Statistical analysis

Mann–Whitney and One-way tests were used to evaluate differences between two or more groups, respectively. Paired samples *t*-test was applied to compare the mean level of expression within the same specimens. Overall survival was analyzed using the Kaplan–Meier estimator, and the differences were evaluated using the log-rank test. This survival analysis was performed to determine the survival of all patients according to the levels of CD8 positive or negative NKs. The Spearman’s Rank test and principal component analysis were used to determine the relationship between different variables. Receiver operator characteristic curve (ROC) analysis was conducted to calculate the area under the curve (AUC). To calculate the ROC curve, all patients were used and compared those with CR compared to the rest of the patients in the study. Uni- and multivariate Cox regression analyses were used with all study patients to estimate hazard ratios (HRs) and the influence of CD8 positive and negative NK variables on survival time, independent of treatment response. All statistical analyses in the study were performed using the software’s GraphPad Prism (6.01), JMP (V.10), and SPSS (V.25.0). The average of samples with SD is presented in all experiments. For all analyses, *p*-values of ≤0.05 were considered statistically significant.

## Results

### Baseline clinical characteristics of R/R DLBCL patients

A total of 78 patients were finally considered in the ITT analysis. In terms of clinical characteristics, the median age was 66 (range 23-86) years, and of the total number of patients 41 were male (51.9%) and 38 female (48.1%). Thirty-three patients (41.8%) were primary refractory DLBCL defined as in the SCHOLAR-1 study (23), and thirty-six samples (64.3%) were classified as non-germinal center B-cell (GCB) and 20 (35.7%) samples as GCB subtype, by Hans algorithm. Of the total patients included, 29 (36.7%) achieved CR, 18 (22.8%) patients had PR, 6 (7.6%) had SD and 25 (31.6%) showed PD. The main baseline characteristics of the patients are summarized in **Supplementary Table 1**. In addition, 10 women and 10 men with a similar median age (68.2 years) to the patients were recruited as healthy donors.

### Blood CD8+ NK level does not change during the treatment of R/R DLBCL patients

**Figure 1:**
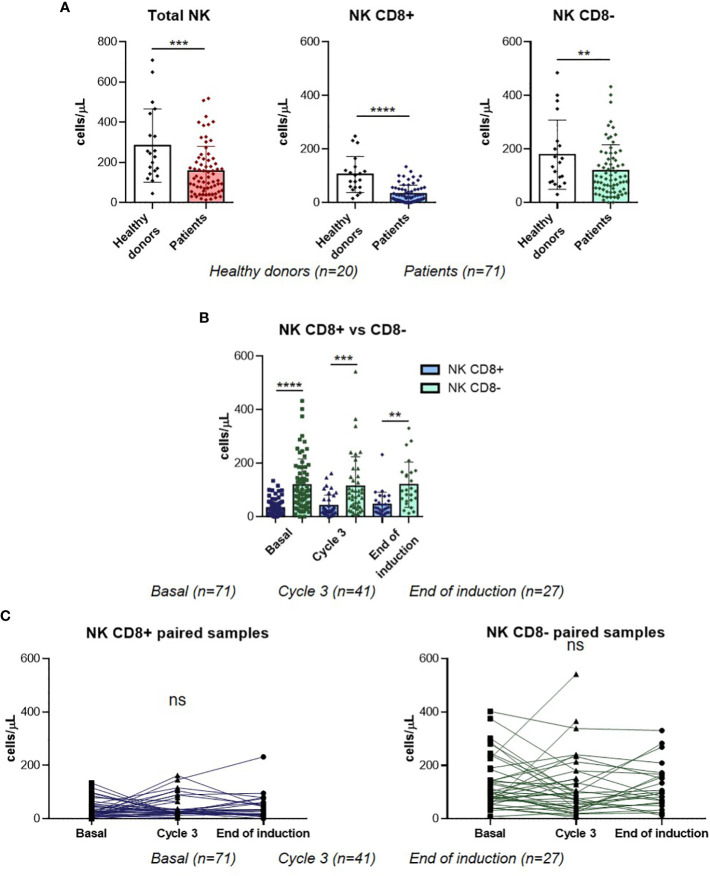
NK populations in R/R DLBCL patients. **(A)** Comparison of total NKs, CD8+ NK and CD8- NK basal levels between healthy donors and R/R DLBCL patients. **(B)** CD8+ NK and CD8- NK levels comparison at three time-points in all samples. **(C)** CD8+ NK and CD8- NK levels comparison at three time-points in paired samples. For all the analyses, *P ≤ 0.05, **P ≤ 0.01, ***P ≤ 0.001 and ****P ≤ 0.0001. ns, not significant.

In order to understand the potential roles of CD8+ NK cell subpopulation, we first compared their basal blood levels in R/R DLBCL patients with those in healthy donors. Circulating CD8+ NK levels were significantly lower in R/R DLBCL patients compared with healthy donors (p<0.0001). Moreover, CD8- NKs and total NKs were also lower in patients (p=0.0271 and p=0.0005 respectively) (**Figure 1A**). Next, both subpopulations were compared before (baseline), during (Cycle 3) and after treatment (EOI) in R/R DLBCL patients, finding that the levels of CD8- NKs were significantly higher than CD8+ NKs levels at all sampling stages (**Figure 1B**). Finally, we studied how the levels of CD8+ NKs evolved during the treatment. There were no differences neither CD8+ NK nor CD8- NK subpopulations before, during and after treatment in paired samples (**Figure 1C**). In addition, the same results were observed when the analyses were with all samples (**Supplementary Figure 2**). In this study, CD8+ NKs are the minority subset of circulating NKs observed in R/R DLBCL patients, and the treatment did not modify their levels.

### High-circulating CD8+ NK levels at baseline were associated with complete response to treatment

Once the number of circulating CD8+ NKs was assessed, we performed a detailed analysis of the number of CD8+ NKs in relation to clinical parameters. No significant differences in CD8+ NK levels were observed between the tumor molecular subtype in DLBCL (GCB and no GCB by Hans algorithm), or between patients with or without refractory disease (**Supplementary Figure 3**). There was also no significant difference neither between elderly and non-elderly patients, nor between men and women (**Supplementary Figure 3**). In addition, results shown in CD8- NKs and total NKs did not change significantly in either of the clinical parameters studied (**Supplementary Figure 3**).

**Figure 2:**
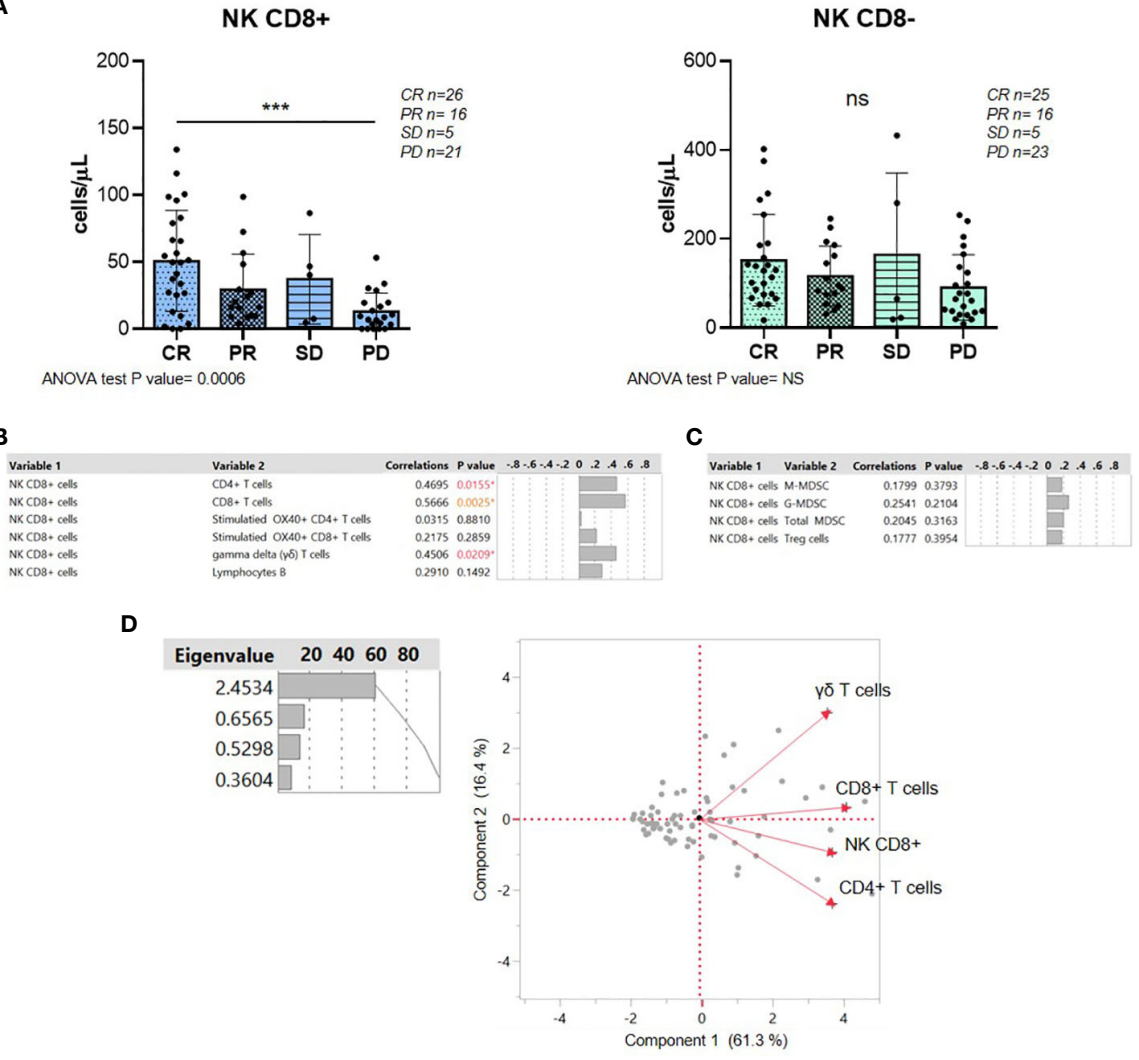
High levels of circulating CD8+ NKs were associated with a complete response to treatment. **(A)** Analysis of CD8+ and CD8- NK levels according to R/R DLBCL patients’ response to treatment. **(B)** Multivariate correlations between CD8+ NKs and protumor immune cells in R/R DLBCL patients with CR. **(C)** Multivariate correlations between CD8+ NKs and antitumor immune cells in R/R DLBCL patients with CR. **(D)** Principal component analysis represented by biplot showed the positive relations between NK CD8+ and CD4+, CD8+ and γδ T cells. For all the analyses, *P ≤ 0.05, **P ≤ 0.01, ***P ≤ 0.001 and ****P ≤ 0.0001. ns, not significant.

When response to treatment was analyzed, R/R DLBCL patients with CR had a significant higher level of basal CD8+ NKs than patients with PD (p=0.0006) (**Figure 2A**), whereas there were no differences in treatment response in CD8- NK subpopulation (**Figure 2A**). To rule out that the result obtained from CD8+ NK cells with response to treatment was based on confounding factors, we performed an analysis between the clinical characteristic of the patients and response to treatment. No association was observed with age, gender, molecular subtypes and refractory disease (**Supplementary Figure 4A**).

Since clinical response was associated with different levels of CD8+ NKs prior to therapy at basal point, we aimed to further analyze the direct relationship of this promising biomarker with response to treatment in these patients. We next performed a multivariate analysis with pro- and anti-tumor immune cells from CR and PD groups. However, in those patients with PD, levels of CD8+ NKs did not correlate with any immune cell studied at baseline (**Supplementary Figures 4B, C**). Instead, a positive correlation was observed between CD8+ NKs and CD4 and CD8+ T cells (p=0.0155 and p=0.0025) and gamma delta (γδ) T cells (CD3+C4-CD8-) (p=0.0209) in patients with CR before treatment (**Figure 2B**). Nevertheless, there was no correlation with other antitumor immune cells such as activated T cells or the immunosuppressive MDSCs (**Figures 2B, C**). Principal component analysis in CR patients also showed a positive relationship between CD8+ NKs, CD8+ and γδ T cells (**Figure 2D**). Together, high-circulating CD8+ NK levels at baseline are associated with CR in R/R DLBCL patients. In addition, in those CR patients, the CD8+ NK subpopulation correlates with the antitumor immune response.

### CD8+ NK cells are potential predictive and prognostic factors to R2-GDP treatment in R/R DLBCL patients

**Figure 3:**
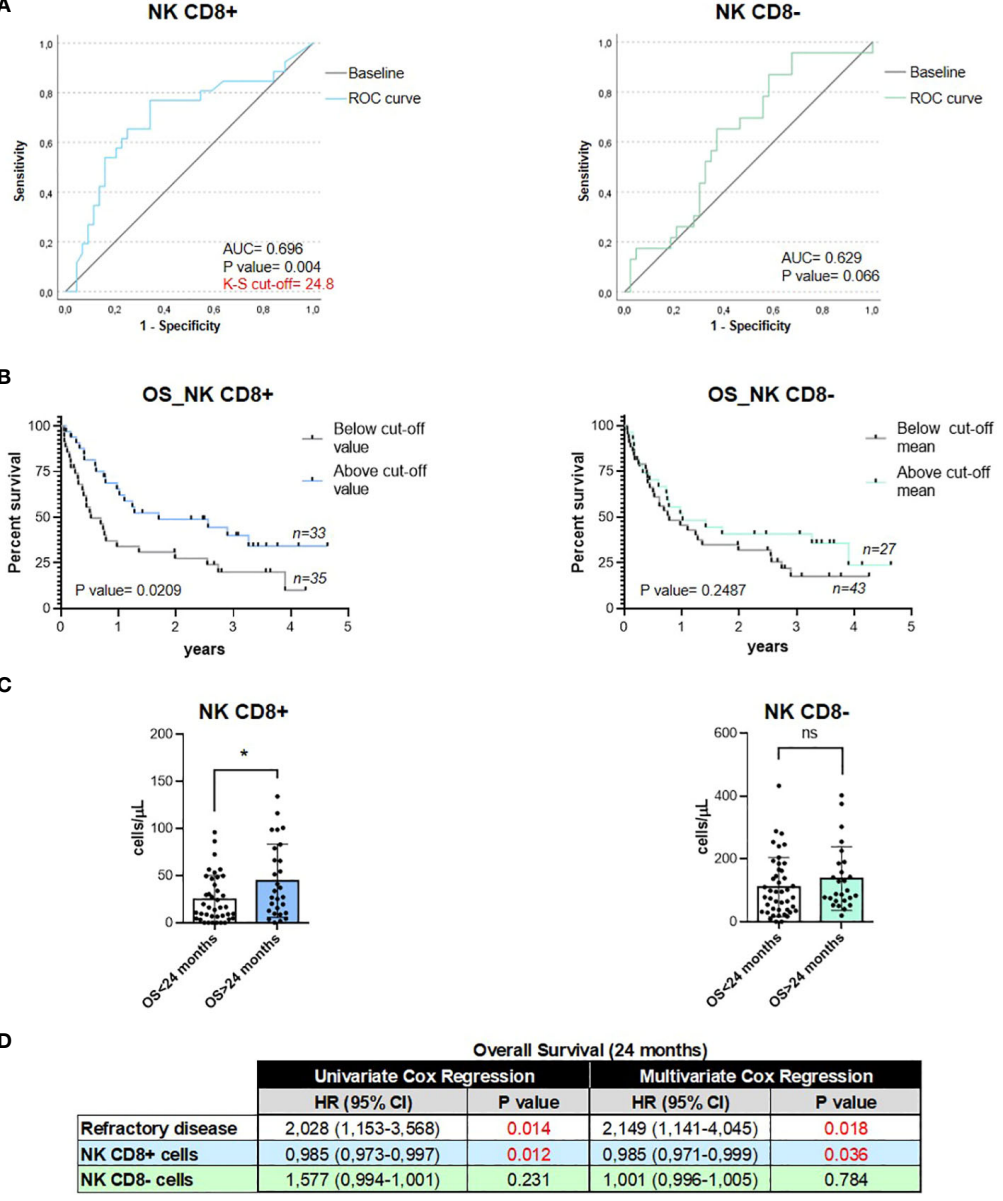
Circulating CD8+ NKs were a prognostic value for predicting the response to treatment and overall survival of R/R DLBCL patients. **(A)**. ROC curve analyses of both subsets of NKs at baseline for the prognosis of treatment response in R/R DLBCL patients. **(B)**. Kaplan–Meier curves of overall survival according to the levels of circulating CD8+ NKs and CD8- NKs in R/R DLBCL patients. **(C)**. Levels of CD8+ NKs and CD8- NKs in patients with OS >24 months and OS <24 months. **(D)**. Cox-regression analysis of CD8 NK cells subpopulations and primary refractory disease in patients. For all the analyses, *P ≤ 0.05, **P ≤ 0.01, ***P ≤ 0.001 and ****P ≤ 0.0001. ns, not significant.

Given the CD8+ NK association with treatment response, we investigated the prognostic potential at baseline in lymphoma patients. The area under the curve (AUC) of CD8+ and CD8- NKs was 0.698 (95% confidence interval [CI], 0.563–0.833; p=0.004) and 0.629 (95% CI, 0.491–0.766; p=0.066), respectively (**Figure 3A**). In the CD8+ NK subset, the cutoff value of the predictive score at the optimum point was 24.8, the specificity was 34.1%, and the sensitivity was 76.9% (**Supplementary Table 4**). Therefore, ROC curve analyses indicated that CD8+ NK levels at baseline are a good predictor of treatment response in R/R DLBCL patients but not the CD8- NK subset. Then, we analyzed the relationship between OS and CD8+ NK levels by Kaplan–Meier test using the ROC curve cutoff level 24.8. We observed that patients with high levels of CD8+ NKs showed a higher survival rate than patients with low CD8+ NK levels (p=0.0209) (**Figure 3B**). However, there were no differences in survival in CD8- NK population (p=0.2487) (**Figure 3B**). In addition, two groups were performed, both for CD8+ NKs and CD8- NKs, depending on whether the patients had a longer or shorter survival than 24 months. Significant high levels of CD8+ NK subset at baseline were found in those patients with an OS >24 months compared with those patients with an OS <24 months (p=0.0142); whereas there were no changes in OS at 24 months in CD8- NKs (p=0.0974) (**Figure 3C**). Finally, we also examined the prognostic role of CD8+ NKs by Cox-regression analysis of OS. Here, the patients with primary refractory disease (a critical prognosis factor in DLBCL patients) and the levels of two CD8 NK subpopulations were explored. Uni- and multivariate analysis showed that only CD8+ NK was strongly associated with OS in an independent manner to the primary refractory disease (p=0.012 and p=0.036, respectively) (**Figure 3D**). These results indicate that the basal levels of circulating CD8+ NKs can be used as an emerging non-invasive and independent biomarker with prognostic and predictive potential.

## Discussion

NKs are cytotoxic lymphocytes with an important antitumor function acting as the first line of defense in tumor surveillance. These effector cells exert natural cytotoxicity against tumor cells by inhibiting their proliferation, migration, or colonization of distant tissues (24, 25). Thus, understanding the role of innate immunity in cancer, and NKs, in particular, is attracting increasing attention. This has led to the discovery of various NK subsets with different immune functions such as CD8+ NKs. We hypothesized that circulating CD8+ NKs may provide a potential information regarding disease progression in R/R DLBCL patients. To elucidate the role of CD8+ NKs in peripheral blood in R/R DLBCL patients, we first observed that the levels of the two subtypes are different, with higher percentage of CD8- NKs. Moreover, a significant decreased number of circulating CD8+ NKs as well as in CD8- NKs and total NKs was found in R/R DLBCL patients compared to healthy donors. These observations are in line with other studies in cancer in which a decrease in the NK cell numbers is often reported (26, 27). However, before, during and after treatment the levels of both CD8 NK subsets did not change, which are in concordance with Waidhauser J. et al., who also showed no significant changes before and after chemotherapy in the count of NKs in solid tumors (28). Thus, no influence of treatment on the CD8 NK subsets may be identified.

Then, we addressed the question whether the peripheral blood profiles of both NK (CD8- and CD8+) populations were correlated with clinical status. Although both subsets of NKs were clearly altered in R/R DLBCL patients as compared to healthy controls, no major shifts were observed in relation to tumor phenotype, refractory disease, gender, or age of patients. However, differences in NK subsets at baseline were related to treatment response. Those patients with CR had significant higher basal levels of CD8+ NKs than those patients in which the disease progressed, whereas no differences in treatment response in CD8- NK subpopulation were observed. In patients with DLBCL, peripheral blood NKs (CD3-CD16+ and/or CD56+) count was associated with treatment response (7). In this study, responders to induction treatment (complete remission, uncertain complete remission or partial remission) had higher levels of total NKs than non-responders (7). To the best of our knowledge, this is the first time that CD8+ NKs demonstrate its potential value as a predictive factor for treatment response in cancer.

In addition to the relationship between CD8+ NKs and response to treatment, we also evidenced a positive interaction between CD8+ NK and some circulating immune cell in responder patients, but not in those patients that progressed on treatment. NKs play a role in antitumor immunity because of both their cytotoxic capacity and their ability to modulate the immune response. Indeed, through cytokines and chemokines production, NKs impact the function of B and T cells responses, dendritic cells, macrophages or neutrophils (29) hindering tumor cell growth, whereas MDSCs promote tumor growth and progression (30, 31). In fact, it has been described that circulating NKs were positively correlated to T and B lymphocytes in cancer (27). Therefore, the correlation with only immune cells that also hinder tumor cell growth in patients with CR would suggest the presence of an antitumor environment that induce a better response to treatment in these patients.

Given that CD8 expression in NKs implies a higher cytotoxic activity (14–16, 18, 19) and they are associated with slower disease progression in HIV patients (17), the prognostic potential of CD8+ NK at baseline were explored. The analyses showed that CD8+ NKs are useful as biomarkers regarding the treatment response in R/R DLBCL patients. In addition, patients with higher levels of CD8+ NKs showed a higher survival rate than patients with low CD8+ NK levels. Since patients in this clinical trial were treated with anti-CD20 (rituximab), which has high affinity for Fc gamma receptors, including FcγRIIIa (expressed on the surface of NKs), clinical results may be explained at least in part by an enhanced FcγRIIIa-mediated ADCC. In this context, recent data of treatment of DLBCL patients with tafasitamab (anti-CD19) and lenalidomide demonstrated an enhanced NK-cell–mediated antibody ADCC by tafasitamab *in vitro* (32–34). We have found that treatment with anti-CD20 and lenalidomide is very effective in those patients with higher level of CD8+ NKs. Therefore, a possible mechanism of treatment response may be the increased ADCC in this subpopulation of NKs (CD8+) potentiated by lenalidomide, what could also partially elucidate the immune effects and mechanism of action of lenalidomide and other immunomodulatory drugs (IMIDs). Nevertheless, this mechanism remains speculative and further studies are needed to confirm this hypothesis. Besides, new therapeutic choices in R/R DLBCL, including NK-CART, reinforces the value and potential clinical applicability of our results.

Finally, in the light of the multivariate analysis, the significant correlation between OS and CD8+ NKs was independent of other relevant clinical parameters such as primary refractory disease, which may point to CD8+ NKs as a molecular factor with a relevant prognostic value in DLBCL.

In conclusion, the search for immune biomarkers is critically important for identifying patients who may be more likely to benefit from cancer therapies. Nevertheless, the discovery of new biomarkers poses challenges, as integrating new biomarkers into clinical practice effectively and accurately in daily practice is a challenge. In line with this, we previously found in this clinical trial that circulating MDSCs along the course of antineoplastic treatment are a promising biomarker in the clinical management of these patients (2, 21), although these immune cells could not predict survival or clinical benefit measured before treatment. Here instead we have demonstrated that the number of circulating CD8+ NKs at baseline is a favorable predictor of survival outcomes and complete response to treatment in patients with R/R DLBCL. Therefore, it could be used as a potential non-invasive predictive and prognostic biomarker. Moreover, our study reveals the existence of novel NK cell subsets displaying different functions in R/R DLBCL patients. Finally, the identification of CD8+ NKs as a unique marker in this tumor may represent an important advance in our understanding of lymphomas, especially DLBCL. Further studies are required to validate this potential biomarker, and to be analyzed in other lymphoma subtypes, due to its biological heterogeneity, and in other cancer types.

## Author’s note

Disclosures provided by the authors are available with this article at doi: 10.1158/1078-0432.CCR-22-0588.

## Data availability statement

The original contributions presented in the study are included in the article/**Supplementary Material**. Further inquiries can be directed to the corresponding authors.

## Ethics statement

The studies involving humans were approved by Seville Provincial Ethics Committee for Research with Drug. The studies were conducted in accordance with the local legislation and institutional requirements. The participants provided their written informed consent to participate in this study. Written informed consent was obtained from the individual(s) for the publication of any potentially identifiable images or data included in this article.

## Author contributions

LH-P: Conceptualization, Data curation, Formal Analysis, Investigation, Supervision, Writing – original draft. DG-D: Conceptualization, Formal Analysis, Investigation, Supervision, Visualization, Writing – original draft. NP-C: Investigation, Writing – review & editing. AG-S: Investigation, Writing – review & editing. EN-F: Investigation, Writing – review & editing. CJ-C: Investigation, Writing – review & editing. MS-L: Investigation, Writing – review & editing. SS-R: Investigation, Validation, Writing – review & editing. RF-C: Investigation, Visualization, Writing – review & editing. FC-G: Investigation, Writing – review & editing. ER-H: Investigation, Writing – review & editing. FC-V: Investigation, Writing – review & editing. GR-G: Investigation, Writing – review & editing. RF-Á: Investigation, Writing – review & editing. NM-B: Investigation, Writing – review & editing. JG-P: Investigation, Writing – review & editing. JG-C: Investigation, Writing – review & editing. AS-S: Investigation, Writing – review & editing. DR-A: Investigation, Writing – review & editing. LG-C: Investigation, Writing – review & editing. JL: Investigation, Writing – review & editing. MG-R: Investigation, Writing – review & editing. MP-P: Investigation, Writing – review & editing. MS-B: Investigation, Writing – review & editing. LM: Investigation, Writing – review & editing. TÁ-N: Investigation, Writing – review & editing. MC-E: Investigation, Writing – review & editing. AR-D: Investigation, Writing – review & editing. VS-M: Conceptualization, Formal Analysis, Investigation, Supervision, Writing – original draft. LC-M: Writing – original draft, Funding acquisition, Investigation, Supervision, Writing – original draft.
