# Supplementary material for: CD8+ NKs as a potential biomarker of complete response and survival with lenalidomide plus R-GDP in the R2-GDP-GOTEL trial in recurrent/refractory diffuse large B cell lymphoma
Source: Front Immunol. 2024 Feb 26;15:1293931. doi: 10.3389/fimmu.2024.1293931 (PMC10926187; doi:10.3389/fimmu.2024.1293931)
Supplement: Supplementary file 1 [file DataSheet_1.pdf]

SUPPLEMENTAL FIGURES

A

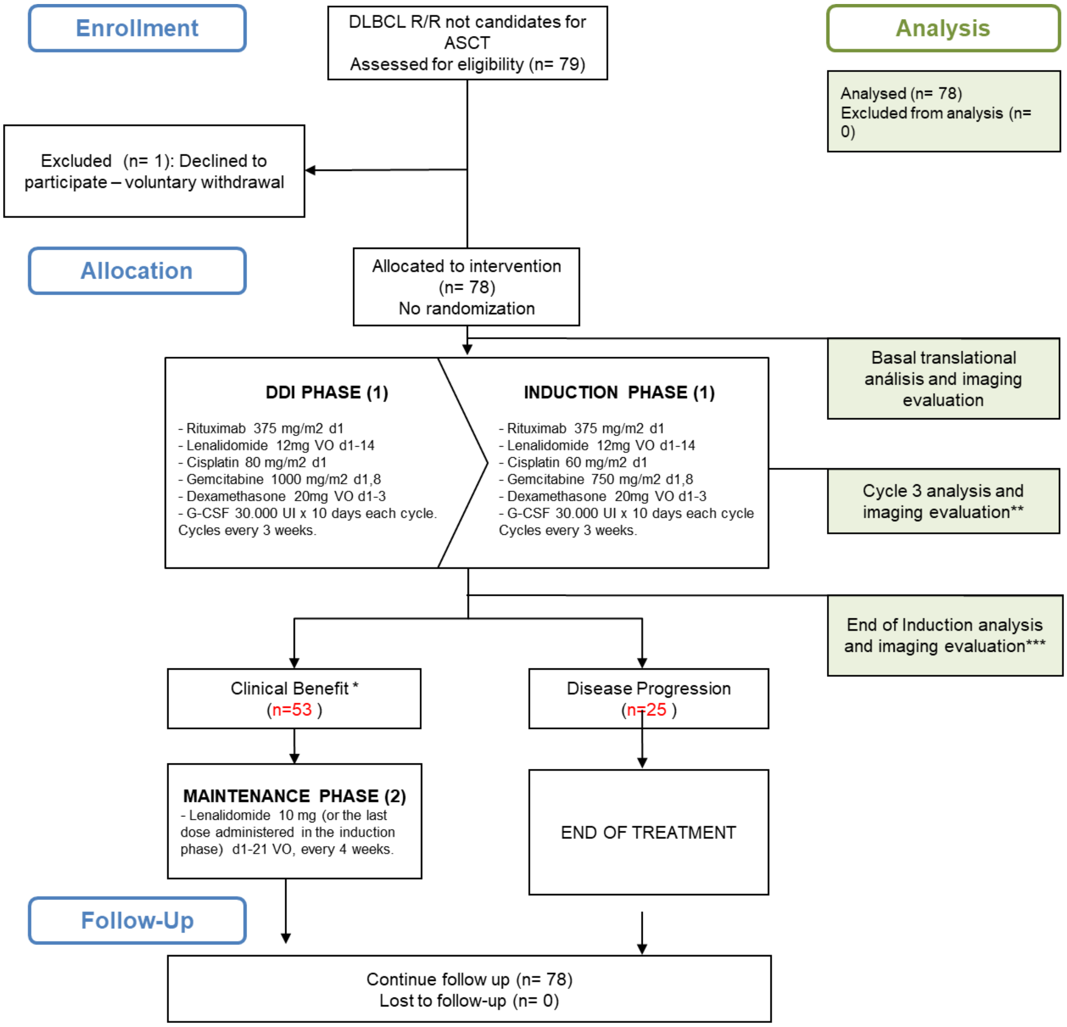

* Clinical Benefit understood as complete response (CR), partial response (PR) or stable disease (SD).
** CT scan
*** PET scan
(1) If after the 3rd cycle there was no progression of disease, a maximum of 6 induction cycles were administered.
(2) Patients that reached clinical benefit after at least 3 cycles of induction phase of treatment could enter a maintenance phase.

B

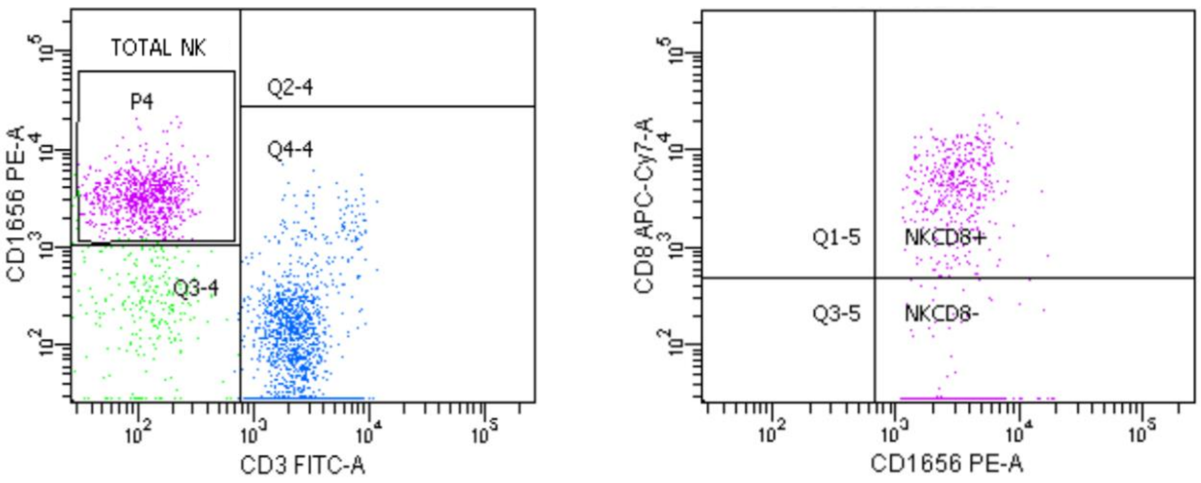

Figure S1: A. Consort Flow Diagram. **B**. Gating of CD8+ NK populations by flow cytometry.

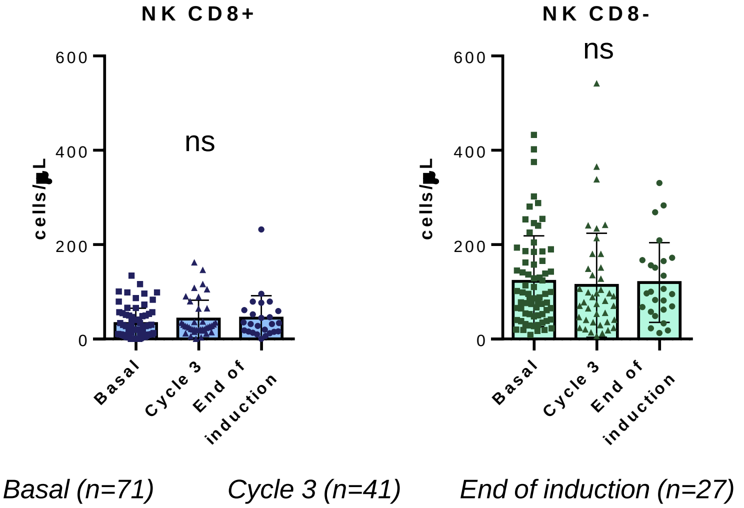

Figure S2: Study of both subpopulations before (baseline), during (Cycle 3) and after treatment (End of induction) in all R/R DLBCL patients.

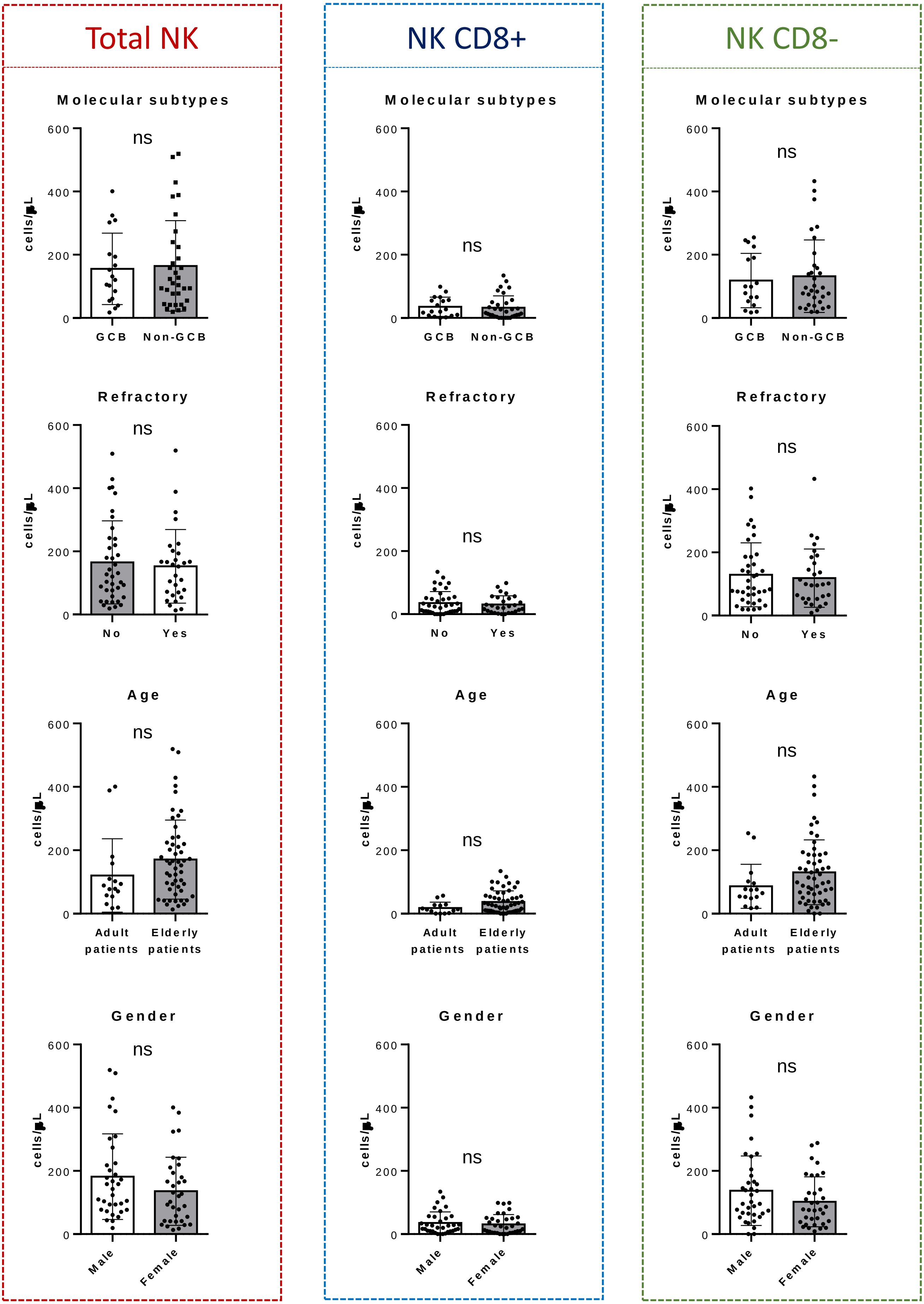

Figure S3: Analysis of the number of total NKs, CD8+ NKs and CD8- NKs in relation to clinical parameters.

A

Variable 1	Variable 2	Correlations	P value
RESPOND TO TREATMENT	AGE	-0.1269	0.2684
RESPOND TO TREATMENT	GENDER	0.0809	0.4813
RESPOND TO TREATMENT	REFRACTARY DISEASE	0.1856	0.1038
RESPOND TO TREATMENT	MOLECULAR SUBTYPES	-0.0940	0.4907

B

Variable 1	Variable 2	Correlations	P value
NK CD8+ cells	CD4+ T cells	0.3913	0.0649
NK CD8+ cells	CD8+ T cells	0.2920	0.1764
NK CD8+ cells	Stimulatied OX40+ CD4+ T cells	0.0030	0.9892
NK CD8+ cells	Stimulatied OX40+ CD8+ T cells	-0.0556	0.8010
NK CD8+ cells	gamma delta (γδ) T cells	0.2175	0.3187
NK CD8+ cells	Lymphocytes B	0.2403	0.2694

C

Variable 1	Variable 2	Correlations	P value
NK CD8+ cells	M-MDSC	0.3893	0.0664
NK CD8+ cells	G-MDSC	0.0258	0.9069
NK CD8+ cells	Total MDSC	0.3555	0.0960
NK CD8+ cells	Treg cells	0.0685	0.7561

Figure S4: A. Multivariate correlations between response to treatment and clinical parameters in all R/R DLBCL patients. **B.** Multivariate correlations between CD8+ NKs and protumor immune cells in R/R DLBCL patients with PD. **C.** Multivariate correlations between CD8+ NKs and antitumor immune cells in R/R DLBCL patients with PD.

SUPPLEMENTAL TABLE

Supplemental Table 1. Summary of clinical characteristics of R/R DLBCL patients.

**Characteristics**	
Patients	79
Median age (range, years)	66.9 (23–86)
**Gender**	
Male	41 (51.9%)
Female	38 (48.1%)
**Disease**	
Refractory	33 (41.8%)
Relapse (non-refractory)	46 (58.2%)
**Molecular subtypes**	
GCB	20 (25.3%)
Non-GCB	36 (45.6%)
Not determined	23 (29.1%)
**Response to treatment**	
Complete response (CR)	29 (36.7%)
Partical response (PR)	18 (22.8%)
Stable disease (SD)	6 (7.6%)
Progresion of the disease (PD)	25 (31.6%)
Not determined	1 (1.3%)

Supplemental Table 2. Monoclonal antibodies used in immunophenotyping for R/R DLBCL patients.

Monoclonal antibodies	Reference
PerCP-Cy5.5 Mouse Anti-Human CD45	no. 564105
APC-Cy7 Mouse Anti-Human CD3	no. 560176
TBNK (including CD3 FITC / CD16 PE + CD56 PE / CD45 PerCP-Cy™5.5 / CD4 PE-Cy™7 / CD19 APC / CD8 APC-Cy™7)	no. 644611
PE-Cy7 Mouse Anti-Human CD4	no. 557852
PerCP-Cy5.5 Mouse Anti-Human CD8	no. 565310
APC-Cy7 Rat Anti-CD11b	no. 557657
PE Mouse Anti-Human CD33	no. 555450
FITC Mouse Anti-Human CD14	no. 555397
APC Mouse Anti-Human CD15	no. 551376
PE-Cy7 Mouse Anti-Human HLA-DR	no. 560651
FITC Mouse Anti-Human CD134 (OX40)	no.555837
APC Mouse Anti-Human PD-1 (CD279)	no. 558694
Human regulatory T cell cocktail (including PerCP Mouse Anti-Human CD4, PE Mouse Anti-Human CD127, and FITC Anti-Human CD25)	no. 560249

Supplemental Table 3. Cell surface marker phenotype of each immune cell subset assessed by flow cytometry.

Immune cells	Phenotype
**Anti-tumoral cells**	
Total NK	CD3- CD16+ CD56+
NK CD8+	CD3- CD16+ CD56+ CD8+
NK CD8-	CD3- CD16+ CD56+ CD8-
Lymphocytes T	CD3+ CD4+
	CD3+ CD8+
	CD3+ CD4- CD8-
Lymphocytes B	CD19+
Stimulatied OX40+ T cells	CD3+ CD4+ OX40+ PD-1-
	CD3+ CD8+ OX40+ PD-1-
**Pro-tumoral cells**	
M-MDSCs	CD45+ CD11b+ CD33+ HLA-DR$^{low/–}$ CD14+ CD15-
G-MDSCs	CD45+ CD11b+ CD33 HLA-DR$^{low/–}$ CD14- CD15+
Tregs	CD4+ CD25^{high} CD127$^{low/–}$
**Total Leukocytes**	CD45+

Supplemental Table 4. Sensitivity and specificity of ROC curve analysis in CD8+ NKs.

Coordinates of the ROC Curve NK CD8+

Test Result Variable(s):

Positive if Greater Than or Equal To[a]	Sensitivity	1 - Specificity
-1.0000	1.000	1.000
0.8500	0.923	0.886
2.4500	0.885	0.886
3.4000	0.885	0.864
3.7000	0.885	0.841
4.0000	0.846	0.841
4.3000	0.846	0.818
4.6000	0.846	0.795
5.6500	0.846	0.773
6.7000	0.846	0.750
7.2500	0.846	0.727
8.1500	0.846	0.705
8.7500	0.846	0.682
8.9000	0.846	0.659
9.3000	0.846	0.636
10.0000	0.808	0.591
10.6000	0.808	0.568
11.7500	0.808	0.545
13.2000	0.769	0.545
14.6000	0.769	0.523
15.8500	0.769	0.500
16.5000	0.769	0.455
18.2000	0.769	0.432
19.7000	0.769	0.409
20.0000	0.769	0.386
22.2500	0.769	0.364
24.8000	0.769	0.341
26.0500	0.731	0.341
27.0000	0.692	0.341
27.3000	0.654	0.341
28.1000	0.654	0.318
29.2000	0.654	0.295
30.0000	0.654	0.273
32.1000	0.654	0.250
33.8500	0.615	0.250
35.5500	0.615	0.227
38.7500	0.577	0.227
40.6500	0.577	0.205
43.9000	0.538	0.205
47.6000	0.538	0.182
48.9500	0.538	0.159
49.6000	0.500	0.159
50.5500	0.462	0.159
52.3000	0.423	0.159
53.9000	0.423	0.136
55.5500	0.385	0.136
56.6000	0.346	0.136
61.2000	0.346	0.114
66.0500	0.308	0.114
69.5000	0.269	0.114
75.8000	0.269	0.091
81.0000	0.231	0.091
84.7500	0.192	0.091
91.2500	0.192	0.068
97.3500	0.154	0.068
99.7500	0.115	0.045
108.5000	0.077	0.045
125.1000	0.038	0.045
134.6000	0.000	0.045
147.7000	0.000	0.023
161.2000	0.000	0.000

The test result variable(s): NK CD8+ has at least one tie between the positive actual state group and the negative actual state group.

a. The smallest cutoff value is the minimum observed test value minus 1, and the largest cutoff value is the maximum observed test value plus 1. All the other cutoff values are the averages of two consecutive ordered observed test values.

Coordinates of the ROC Curve NK CD8-

Test Result Variable(s):

Positive if Greater Than or Equal To[a]	Sensitivity	1 - Specificity
16.2000	1.000	1.000
18.2000	0.957	1.000
19.3000	0.957	0.977
19.5000	0.957	0.953
21.2000	0.957	0.930
25.7500	0.957	0.907
29.1500	0.957	0.884
30.5500	0.957	0.860
33.1500	0.957	0.837
36.3000	0.957	0.814
38.1000	0.957	0.791
39.4000	0.957	0.767
40.7500	0.957	0.744
44.5500	0.957	0.721
49.2000	0.957	0.698
51.5500	0.957	0.674
52.8000	0.913	0.674
53.4500	0.870	0.674
57.6000	0.870	0.651
63.0000	0.870	0.628
64.9500	0.870	0.605
65.4000	0.870	0.581
66.4500	0.826	0.581
70.6000	0.783	0.581
74.4000	0.783	0.558
75.5000	0.739	0.558
76.6000	0.696	0.558
77.2500	0.696	0.535
77.7500	0.696	0.512
80.6000	0.696	0.488
84.6500	0.696	0.465
87.0500	0.652	0.465
91.9500	0.652	0.442
97.3500	0.652	0.395
99.2000	0.652	0.372
100.6500	0.609	0.372
106.0500	0.565	0.372
112.0000	0.565	0.349
118.9500	0.522	0.349
126.4000	0.522	0.326
129.3500	0.478	0.326
133.5000	0.435	0.326
137.7000	0.435	0.302
139.8000	0.391	0.302
141.9000	0.348	0.302
144.0000	0.304	0.302
151.4500	0.304	0.279
159.8500	0.261	0.279
163.8000	0.261	0.256
175.2000	0.261	0.233
185.3500	0.261	0.209
186.1000	0.217	0.209
188.1500	0.217	0.186
191.8000	0.174	0.186
199.1000	0.174	0.163
215.1000	0.174	0.140
232.9500	0.174	0.116
243.0000	0.174	0.093
249.6000	0.174	0.070
254.1500	0.174	0.047
267.8000	0.130	0.047
284.4000	0.130	0.023
295.2000	0.087	0.023
338.8000	0.043	0.023
403.8500	0.000	0.023
433.5000	0.000	0.000

a. The smallest cutoff value is the minimum observed test value minus 1, and the largest cutoff value is the maximum observed test value plus 1. All the other cutoff values are the averages of two consecutive ordered observed test values.
